# What drives low-carbon agriculture? The experience of farms from the Wielkopolska region in Poland

**DOI:** 10.1007/s11356-021-17022-3

**Published:** 2021-10-25

**Authors:** Michał Borychowski, Aleksander Grzelak, Łukasz Popławski

**Affiliations:** 1 Department of Macroeconomics and Agricultural Economics, Poznań University of Economics and Business, 61-875 Poznań, Poland; 2 Department of Public Finance, Cracow University of Economics, Kraków, Poland

**Keywords:** Low-carbon agriculture, The Wielkopolska region, Energy, Fertilizers, Productivity, Agricultural efficiency, Structural equation modeling (SEM), O13, Q10, Q15, Q56

## Abstract

Because of global environmental problems, low-carbon agriculture has gained increasing importance both in developed and developing countries. Hence, there is a need to find ways to develop more efficient agricultural systems. The purpose of this article is to identify the drivers of low-carbon agriculture on farms in the Wielkopolska region (in Poland). We aimed to take an original approach to investigate low-carbon agriculture with a unique set of different economic and environmental variables and contribute to the literature, which is not very extensive in terms of microeconomic research, including research on farmers in the Wielkopolska region. Therefore, we employed a multiple-factor measurement model for structural equation modeling (SEM) of data collected individually from 120 farms in 2020. As a result, we formulated the following conclusions: the increasing productivity of factors (land, labor, and capital) have a positive effect on low-carbon farming, just as increasing fertilizer and energy efficiency. Moreover, thermal insulation is also important for low-carbon agriculture, with efficiency of fertilizer use being the most important factor. We believe that the issues of farm use of fertilizers and thermal insulation of buildings should be more broadly included in energy policy, both at the national and the European Union (EU) levels. Some of these factors however are already present in the common agricultural policy (CAP) for 2021–2027.

## Introduction

Climate change has an increasingly significant impact on a number of industries and economic sectors, and mainly refers to seven sectors: energy, industry, agriculture, water and sanitation, transportation, urban development, and tourism (Chen et al. 2020). In agriculture, factory farming is a massive contributor to global warming because of the high methane and nitrous oxide emissions (Rafiq et al. 2016). This leads to an increased interest in low-carbon agriculture, which may be defined as an agricultural system that enables efficient production of the raw materials, food, feed, and fibers while reducing energy inputs and greenhouse gas (GHG) emissions from agriculture and respecting the principles of sustainable development (Piwowar 2019). This means that it is possible to simultaneously achieve both economic (income, economic performance) and environmental (provision of public goods, protection of the environment, internalization of externalities, and preservation of biodiversity) gains. Therefore, a global and local shift in farming can help the transition to a low-carbon economy (Norse 2012). And as Sharma et al. (2021a) emphasized, higher income level easier allows a country to adopt low-carbon practices in the economy (including industry and households). According to Clapp et al. (2010), national plans and strategies for low-emission and climate-resilient economic growth are crucial for sustainable development. However, low-carbon farming requires high annual investments to meet the goals of the Paris Agreement (Van Veelen 2021). Therefore, more attention should be paid to these issues in energy policy. Today, some Polish researchers are investigating measures for reducing the carbon footprint of farming practices. For example, Wiśniewski and Kistowski (2018) analyzed GHG emissions and carbon footprint in Polish agriculture at the local level. In turn, Kistowski and Wiśniewski’s (2020) study puts a particular focus on emissions in the municipalities.

The motivation for this paper is the research gap which exists especially in Central European countries with a medium level of agricultural development in identifying the economic determinants of a low-carbon economy. These problems do not have a clear-cut solution. Researchers have pointed out many factors affecting CO_2_ emissions from agriculture (Zhao et al. 2018). Therefore, the objective of this study was to identify the factors that contribute to the transformation towards low-carbon agriculture in Wielkopolska farms (in Poland). It is worth noting that Poland is one of the largest consumers of mineral fertilizers in Europe (Piwowar 2021). Hence, research in this field seems to be needed and urgent, as Polish agriculture faces several important challenges. The following two hypotheses were adopted: H1: productivity of factors such as land, labor, capital (production factors) positively affects low-carbon farming; H2: increasing fertilizer efficiency and energy efficiency is conducive to low-carbon farming. Our original contribution is that we have taken a unique approach to study low-carbon agriculture (with a set of different economic variables) and employed a well-fitted estimation model for microeconomic data collected from 120 farms in the Wielkopolska region, using structural equation modeling. Structural equation modeling, an extension of the general linear model (StataCorp 2017), provides a more general framework for performing factor analysis. The strength of SEM is that it has all-encompassing possibilities, because it includes path analysis and multivariate regression as special cases. SEM is even more useful when dealing with composite indicators, which is a significant advantage because most simple variables do not sufficiently describe complex theoretical phenomena and concepts. Usually, SEM combines multiple indicators for each latent variable and supports complex paths connecting latent variables (Garson 2015). Moreover, the goodness of the model fit may be verified using many different statistics. The theoretical frameworks for our research are environmental economics and sustainable development (Rogall 2004; Goldin and Winters 2010). Our results may be a basis for formulating recommendations in these areas.

The remainder of the paper is organized as follows: “Literature review” offers a critical review of the literature on carbon emissions, and “Materials and methods” explains the data and methodology. “Results and discussion” describes the results and contains discussion. “Managerial implications” outlines some qualitative managerial implications, while “Conclusion and policy implications” contains conclusions and policy implications.

## Literature review

The scale of global emissions from agriculture has been investigated by numerous studies in recent years (Eyuboglu and Ezar 2020; Groeneveld et al. 2003; Haider et al. 2020; Moran and Knook 2017; Qiao et al. 2019). As emissions concern externalities and international public goods (like the atmosphere), they are of global importance, even though they occur at a local level (Muhammad and Khan 2021). There are also many country-level studies that identify factors affecting CO_2_ emissions in agriculture (Xu and Lin 2017) or drawing attention to the problem of ecological footprint (Alvarado et al. 2021; Khan et al. 2021a; Zia et al. 2021). Interesting studies linked to the relationship between growth processes and carbon emissions are examples of the use of environmental Kuznets curve (Dogan and Seker 2016b; Khan et al. 2021a; Koondhar et al. 2021a; Shahbaz et al. 2020; Sinha and Shahbaz 2018). Other papers highlighted that energy efficiency is linked with the per capita income from the country’s overall economic growth (Zakari et al. 2021). There are also numerous studies that pointed to the impact of financial development (including foreign direct investment) on carbon dioxide (CO_2_) emissions (Ahmad et al. 2019; Muhammad and Khan 2021; Muhammad et al. 2021; Shahbaz et al. 2020, 2021), carbon intensity (Sharma et al. 2021b), and study drawing attention to the fact that increasing financial risk directly reduces CO_2_ (Zhao et al. 2021). In turn, Piwowar (2019) presented theoretical and practical challenges for low-carbon agriculture in Poland. As he pointed out, reducing gas emissions in the agricultural sector requires the introduction of innovative techniques and tools to increase the efficiency of production. Syp and Osuch (2018) adopted an interesting approach to assessing GHG emissions from conventional farms. They argued that there are significant differences in the intensity of the GHG emissions among dairy farms in Poland: the highest emissions were noted in medium-small and medium-large farms.

The higher the degree of agricultural mechanization is, the higher the energy consumption (Eyuboglu and Ezar 2020). Therefore, there are many actions that farmers can take to reduce emissions. These include increasing carbon sequestration in the soil (West and Marland 2002; Norse 2012; Reay 2020; Xiao et al. 2021); reducing the use of mineral fertilizers and replacing them with recycled fertilizers (Kytta et al. 2021), as well as using fertilizer appropriately and decreasing energy intensity (Solinas et al. 2021), and decreasing the use of mineral pesticides while introducing integrated pest management (Piwowar 2021); lowering the overuse and misuse of nitrogen (Norse 2012); reducing energy consumption and increasing energy efficiency (Piwowar 2019); replacing fossil fuels by renewable energy sources (Dogan and Seker 2016a; Eyuboglu and Ezar 2020, Paramati et al. 2018); and improving animal feeding techniques and animal husbandry systems as well as designing and using energy-efficient livestock buildings (Kistowski and Wiśniewski 2020). It is also important to optimize animal nutrition and efficient waste management, which have a positive impact on reducing emissions. A wide range of mitigation technologies and measures was reported and discussed by Eyuboglu and Ezar (2020) and Xiong et al. (2021). The literature also highlights the potential to improve overall productivity by reducing emissions per unit of product in some systems (Johnson et al. 2007). One of the most important measures for reducing CO_2_ emissions in farms is thermal retrofitting of agricultural buildings, which can also lead to reduced energy consumption (Hörndahl 2008). Some studies have also considered the issue of crop fertilization in the context of CO_2_ emissions. They mainly focused on emissions resulting from the production of fertilizers and plant protection products. These problems were investigated, for example, by Bouwman et al. (2002). In turn, Shahbaz et al. (2016), using Portuguese annual data, highlighted that environmental degradation in terms of CO_2_ emissions can be controlled by using energy-efficient technologies.

Within the European Union, the current course of action, especially since the beginning of the twenty-first century, further reinforces the move towards a zero-emission economy in member countries. Numerous studies (Jensen et al. 2019; Xiong et al. 2016; Wang et al. 2020) also highlight the desirable direction of such changes. According to Moran and Knook (2017), some of the CO_2_ reduction measures in agriculture are relatively cheap in comparison to the cost of reducing emissions in other sectors, such as energy or heavy industry. In turn, Akbar et al. (2021) emphasized in their research, based on experiences in China, that changes in agricultural planting structure, agricultural value-added, and agricultural scale management are also important factors affecting agro-ecological growth; thus, they reduce CO_2_ emissions. Piwowar (2020) emphasized the essential role of agricultural biogas in low-carbon and circular economy. What distinguishes our research approach from the previous studies is the use of microeconometric quantitative as well as qualitative data collected from individual farms and employing structural equation modeling.

## Materials and methods

We employed structural equation modeling, which is considered to be one of the best techniques for analyzing interdisciplinary issues, also within environmental economics (Brown 2015; Hooper et al. 2008). Therefore, it may be applied in our research, which examines economic and environmental interdependencies. SEM combines the advantage of analysis of variance, regression, and factor analysis, extending them with the possibility of modeling cause-and-effect relationships using latent variables (Garson 2015; Brown and Moore 2012; OECD 2008). Any researcher using SEM may identify indirect, direct, and total independencies between variables, both latent and observed variables, and between all specified variables (Garson 2015; Anghel et al. 2019). Hoyle (2012), Kline (2011), and StataCorp (2017) provide very broad and valuable descriptions of SEM and different SEM models, while Garson (2015) discusses the many advantages of SEM. We used a multiple-factor measurement model as a type of SEM, where we have a firm idea and clear expectations about the latent factors and variables that most likely load onto each construct. This model is usually used to test hypotheses or theories about the factors or latent variables that are expected to be found (Brown 2015; Hadrich and Olson 2011). In such a model, every hypothesized connection should be logically justified, and there must be a coherent story behind the entire model (Iacobucci 2010).

According to StataCorp (2017), in any given analysis, there are typically several variables of each type. Vectors of the four main variable types are denoted *y*, *x*, *η*, and *ξ*.

The vector of all endogenous variables is:

1 $$Y=\left(\genfrac{}{}{0pt}{}{y}{\eta }\right)$$

The vector of all exogenous variables is:

2 $$X=\left(\genfrac{}{}{0pt}{}{x}{\xi }\right)$$

The vector of all error variables is:

3 $$\varsigma =\left(\genfrac{}{}{0pt}{}{e.y}{e.\eta }\right)$$

Structural equation modeling can fit models of the form (StataCorp 2017):

4 $${Y}_{i}={BY}_{i}+ {\Gamma X}_{i}+{\alpha }_{Y}+{\varsigma }_{i}$$

where *Y*_*i*_ is a vector of latent endogenous variables for unit *i*; *B* = [*β*_*ij*_] is the matrix of coefficients giving the expected effects of the latent endogenous variables (*Y*) on each other; *X*_*i*_ is the vector of exogenous variables; *Γ* = [*γ*_*ij*_] is the matrix of coefficients on exogenous variables; *α* = [*α*_*Y*_] is the vector of intercepts for the endogenous variables; ${\varsigma }$ is the vector of errors and is assumed to have mean 0 and Cov (*X*, ${\varsigma }$) = 0. Let:

5 $$\kappa =\left[{\kappa }_{ij}\right]=E(X)$$

6 $$\varphi =\left[{\varphi }_{ij}\right]=Var(X)$$

7 $$\psi =\left[{\psi }_{ij}\right]=Var({\varsigma })$$

where *κ* is the vector of latent exogenous means; *φ* is the matrix of latent exogenous variances and covariances; *ψ* is the matrix of latent endogenous error variances and covariances.

Then, the mean vector of the endogenous variables is:

8 $$\mu Y=E\left(Y\right)={(I-\mathrm{B})}^{-1} (\Gamma \kappa +\alpha )$$

The variance matrix of the endogenous variables is:

9 $${\sum }_{YY}=Var\left(Y\right)={\left(I-B\right)}^{-1}({\Gamma \varphi }{\Gamma }^{{^{\prime}}}+\uppsi )\left\{{\left(I-B\right)}^{-1}\right\}{^{\prime}}$$

And the covariance matrix between the endogenous variables and the exogenous variables is:

10 $${\sum }_{YX}Cov\left(Y,X\right)={\left(I-B\right)}^{-1}{\Gamma \varphi }$$

Let *Z* be the vector of all variables:

11 $$Z=\left(\genfrac{}{}{0pt}{}{Y}{X}\right)$$

Then, its mean vector is:

12 $$\mu =E\left(Z\right)=\left(\genfrac{}{}{0pt}{}{{\mu }_{Y}}{\kappa }\right)$$

And its variance matrix is:

13 $$\sum =Var\left(Z\right)=\left(\begin{array}{cc}{\sum }_{YY}& {\sum }_{YX}\\ {\sum }_{YX}^{^{\prime}}& \varphi \end{array}\right)$$

In our model, the maximum likelihood (ML) method is applied. We decided to use this technique because it is flexible, helps to provide reliable results, and allows to analyze various kinds of model in an easy, straightforward, and efficient way. Moreover, maximum likelihood is the method which is recommended and used by default in SEM. The estimation method of the ordinary least square (OLS) is identical to maximum likelihood; however, the first one may be used only in the case of normal distribution of residuals. Therefore, ML is more general and flexible. For the BHHH optimization technique (Berndt-Hall-Hall-Hausman algorithm) and when computing observation-level scores, the log-likelihood (logL) for *θ* is computed as:

14 $$logL\left(\theta \right)=-{\sum }_{t=1}^{N}\frac{{w}_{t}}{2}\left[klog\left(2\pi \right)+log\left\{\mathrm{det}({\sum }_{0})\right\}+({z}_{t}-{\mu }_{0}){^{\prime}}{\sum }_{0}^{-1}\left({z}_{t}-{\mu }_{0}\right)\right]$$

where *θ* is a vector of unique model parameters; *w*_*t*_ is corresponding weight value, where *t* = *1*, …, *N*; *k* is the number of observed variables; *Ʃ*_o_ is the submatrix of *Ʃ* corresponding to the observed variables; *z*_t_ is the vector of all observed variables for the *t*th observation; *μ*_o_ is the subvector of *μ* corresponding to the observed variables.

We used the results of a survey carried out in 2020 on a group of 120 agricultural holdings from the Wielkopolska region in Poland. The holdings were selected based on the economic size of the farms (ES)^1^ and the type of farming (TF).^2^ The research tool was an interview questionnaire. Because of the relatively low number of holdings from groups ES1 and ES6 which keep agricultural accounts in Wielkopolska (31 and 9, respectively), farms from groups ES2 to ES5 were selected for the research. The production types included were TF1 and TF5–TF8. A quota selection of the farms was applied. The planned number of the surveyed farms (120) was divided proportionally according to both the economic size (ES2–ES5) and production type (TF1, TF5–TF8), in the group of Wielkopolska farms that keep agricultural accounts according to Farm Accountancy Data Network (FADN). Then, specific numbers of farms were selected for the study using the records of the Institute of Agricultural and Food Economics-National Research Institute in Warsaw. Next, interviewers were assigned to the selected farms.

Wielkopolska is one of 16 voivodships (regions) in Poland. The agricultural area accounts for 11.3% of all of Poland’s area, and the value of agricultural gross output is 17.4% of the total value for Poland (Grzelak et al. 2020). In our study, we focused on drivers of low-carbon agriculture in the Wielkopolska region. We defined low-carbon agriculture using a set of factors, and then we analyzed the relationships between them and different economic variables, including the factors of productivity. The variables were:

- fert_u_eff: fertilizer use efficiency (the ratio of agricultural output in thousands of PLN (Polish złoty)/1000 kg fertilizer used)
- fert_eff: fertilizer efficiency (the ratio of agricultural output in thousands of PLN/expenditure on fertilizers in thousands of PLN)
- nrg_eff: energy efficiency (the ratio of agricultural output in thousands of PLN/expenditure on energy in thousands of PLN)
- land_prod: land productivity (the ratio of agricultural output in thousands of PLN/agricultural area in hectares)
- lab_prod: labor productivity (the ratio of agricultural output in PLN/number of person-hours worked on a farm)
- cap_prod: capital productivity (the ratio of agricultural output in PLN/the value of total assets in PLN),
- inc_share: share of agricultural income in the total income of a household (in %)
- land_val: land value in thousands of PLN (standardized variable)
- income: agricultural income in thousands of PLN (standardized variable)
- therm_ins: thermal insulation of livestock buildings (dummy variable: 1 = yes; 0 = no)
- low_carbon (latent variable): low carbon agriculture as a series of actions to achieve economic (agricultural) goals while respecting the natural environment
- productivity (latent variable): productivity of factors (land, labor, and capital).

It may seem surprising that the group of variables that make up the low-carbon latent variable does not include a measure of livestock production intensity (e.g., the number of large units per hectare), which has a direct impact on GHG emissions. However, it was not possible to generate a well-fitted model when including this variable. The reason could be that livestock density exceeded the level considered harmful for the environment, 2 large units/ha, in only 18% of the farms (Kopiński and Madej 2006). This figure is due to the official maximum annual limit of 170 kg of nitrogen from livestock manure per 1 ha. On the other hand, the median for livestock intensity was 1.1 and in 17% of the surveyed farms, there was no livestock production. Agriculture in the Wielkopolska region is not intensive compared to other EU regions, which is typical for countries with a medium level of development. Therefore, this variable plays a smaller role in this case.

As we employed structural equation modeling with a multiple-factor measurement model, for every construct, one crucial and logically justified observed variable must be a reference variable. In our case, it is fertilizer use efficiency (fert_u_eff) for the low_carbon agriculture (low_carbon) construct and labor productivity (lab_prod) for productivity construct. The coefficients of other variables are interpreted with reference to the selected variable. The descriptive statistics are given in Table 1.

**Table 1:** Descriptive statistics

Abbreviation	Full description	Descriptive statistics			
		Mean	Std. dev	Min	Max
fert_u_eff	Fertilizer use efficiency (the ratio of agricultural output in thousands of PLN/fertilizer use in 1000 kg)	27.67	40.07	3.02	311.08
fert_eff	fertilizer efficiency (the ratio of agricultural output in thousands of PLN/expenditure on fertilizers in thousands of PLN)	22.50	30.03	2.48	233.90
nrg_eff	Energy efficiency (the ratio of agricultural output in thousands of PLN/expenditure on energy in thousands of PLN)	19.00	23.04	0.01	190.49
land_prod	Land productivity (the ratio of agricultural output in thousands of PLN/utilized agricultural area in ha)	8.56	6.96	0.94	58.97
lab_prod	Labor productivity (the ratio of agricultural output in PLN/number of person-hours worked in a farm)	55.81	47.59	5.67	360.05
cap_prod	Capital productivity (the ratio of agricultural output in PLN/the value of total assets in PLN)	0.17	0.11	0.02	0.57
inc_share	Share of agricultural income in the total income of a household (in %)	76.2%	27.5%	10%	100%
land_val	Land value in thousands of PLN (standardized variable)	969.53	896.20	0	7000
income	Agricultural income in thousands of PLN (standardized variable)	82.39	90.90	− 14.3	439.03
therm_ins	Thermal insulation of livestock buildings (dummy variable: 1 = yes; 0 = no)	Distribution: yes: 20.8%; no: 79.2%			
low_carbon	Low-carbon agriculture as a series of actions to achieve economic (agricultural) goals while respecting the natural environment	Latent variable			
productivity	Productivity of factors (land, labor, and capital)	Latent variable			

Descriptive statistics for variables “land value “ and “income “ are for unstandardized solutions. Source: Elaboration based on own data set

**Fig. 1:**
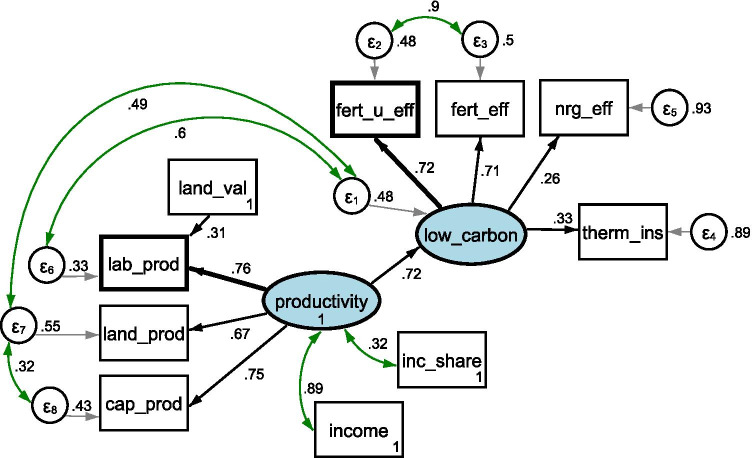
**Drivers of low-carbon farming in the Wielkopolska region: Evidence based on structural equation modeling using a multiple-factor measurement model with latent variables**. Variables in blue ovals (“low_carbon” and “productivity”) are unobserved exogenous latent variables. Variables in rectangles are observed endogenous variables. Moreover, variables in wider rectangles are reference variables (“fert_u_eff” for construct “low_carbon” and “lab_prod” for construct “productivity”). *ε* (in small circles) are errors. The values in the rectangles for observed variables are standardized intercepts. The values on the black arrows between variables are standardized path coefficients (the first column “Coefficient” in table with detailed results). The values on green bidirectional arrows between two observed variables (between errors) or between observed variables and construct are standardized covariances, which are correlation coefficients (the column “Coefficient” in table with detailed results). All values presented in the figure are standardized values in standard deviation units. All variables and covariances are statistically significant (*α* = 0.05)

The econometric strategy was as follows:

- We formulated research goals and hypotheses on the drivers of low-carbon agriculture in Wielkopolska region, namely, H1: factor productivity (land, labor, and capital) positively affects low-carbon farming; H2: increasing fertilizer efficiency and energy efficiency is conducive to low-carbon farming.
- Taking into account that different economic factors may be interconnected, we hypothesized and tested additional covariances and interactions.
- We used structural equation modeling with multiple-factor measurement to determine if the hypothesized relationships hold true. As one of the variables is a dummy variable (thermal insulation), we used a polychoric correlation matrix (instead of a raw database). All estimation was done using STATA 15 software.
- After estimating the model, we checked the model fit Table 3).
- The final model is presented as a graph (Fig. 1), and full results are shown in Tables 2 and 3.

**Table 2:** Results of structural equation modeling using multiple-factor measurement model

Standardized		Coefficient	OIM Std. Err	z	*P* > *z*	95% Conf. interval	
**Structural**							
lab_prod	land_val	0.3137011	0.0486883	6.44	0.000	0.2182739	0.4091284
	productivity	0.7586774	0.0393805	19.27	0.000	0.6814931	0.8358617
low_carbon	productivity	0.7234158	0.110969	6.52	0.000	0.5059206	0.940911
**Measurement**							
fert_u_eff	low_carbon	0.720979	0.0915787	7.87	0.000	0.5414881	0.9004699
fert_eff	low_carbon	0.7098853	0.0948689	7.48	0.000	0.5239456	0.8958249
nrg_eff	low_carbon	0.2573268	0.1035811	2.48	0.013	0.0543115	0.4603421
therm_ins	low_carbon	0.3262237	0.0899785	3.63	0.000	0.1498691	0.5025783
land_prod	productivity	0.6726752	0.0514037	13.09	0.000	0.5719257	0.7734246
cap_prod	productivity	0.7521781	0.0437803	17.18	0.000	0.6663704	0.8379859
var(e.fert_u_eff)		0.4801893	0.1320526			0.2801116	0.8231782
var(e.fert_eff)		0.4960629	0.1346921			0.2913511	0.8446113
var(e.nrg_eff)		0.9337829	0.0533084			0.8349337	1.044335
var(e.therm_ins)		0.8935781	0.0587062			0.785616	1.016377
var(e.lab_prod)		0.3260002	0.0557035			0.2332241	0.4556824
var(e.land_prod)		0.5475081	0.069156			0.4274397	0.7013039
var(e.cap_prod)		0.4342281	0.0658611			0.3225616	0.5845519
var(e.low_carbon)		0.4766695	0.1605534			0.2463275	0.9224056
var(productivity)		1					
cov(e.fert_u_eff,e.fert_eff)		0.902224	0.0291111	30.99	0.000	0.8451673	0.9592807
cov(e.lab_prod,e.low_carbon)		0.603444	0.1821972	3.31	0.001	0.2463441	0.960544
cov(e.land_prod,e.cap_prod)		0.324114	0.0879193	3.69	0.000	0.1517953	0.4964326
cov(e.land_prod,e.low_carbon)		0.4909387	0.1499265	3.27	0.001	0.1970881	0.7847893
cov(income,productivity)		0.887542	0.0297074	29.88	0.000	0.8293166	0.9457675
cov(inc_share,productivity)		0.3223527	0.0543617	5.93	0.000	0.2158058	0.4288997

All abbreviations are explained in Table 1 with descriptive statistics significance level: *α* = 0.05

**Table 3:** Goodness of fit of the estimated model

Measure	*p* > chi^2^	RMSEA	CFI	TLI	SRMR	CD	AIC	BIC
Model	0.450	0.009	1.000	0.999	0.056	0.845	2759.083	2820.408
Threshold value	> 0.05	< 0.08	≥ 0.9 (0.95)	≥ 0.95	< 0.08	The highest possible	The lowest possible	

*RMSEA*, root mean squared error of approximation; *CFI*, comparative fit index; *TLI*, Tucker-Lewis index; *SRMR*, standardized root mean squared residual; *CD*, coefficient of determination; *AIC*, Akaike’s information criterion; *BIC*, Bayesian information criterion

Thresholds value according to Brown (2015); Hooper et al. (2008); Parry (2021)

## Results and discussion

Our estimated model (Fig. 1) meets all criteria for acceptable model fit, including RMSEA: 0.009, CFI: 1.0, TLI: 0.999, SRMR: 0.056, and CD: 0.845. Detailed data is presented in Table 3.

The low_carbon latent variable is most significantly determined by the efficiency of fertilizer use. Much lower significance occurred for the thermal insulation of livestock buildings variable. This resulted from the relatively low prevalence of thermal insulation of buildings in the Polish countryside (23% of farms). However, this is going to change due to the growing popularity of solutions that increase energy efficiency, which in turn contributes to development of low-carbon farming. The lower impact of energy efficiency on the low_carbon construct may be caused by the relatively low level of agricultural development in Poland, which means that its energy intensity is not high. In turn, Rafiq et al. (2016) investigated the impact of sectoral production allocation, energy use patterns, and trade openness on pollutant emissions in a group of 53 countries, including European countries like Bulgaria, Italy, and Portugal, whose agricultural sectors share some characteristics with Poland. They found that energy intensity is a driver of pollution emissions, hence, increasing energy efficiency promotes low-carbon agriculture. Khan et al. (2021b) also pointed out the role of decreasing energy intensity, as well as adopting environmental-related technologies and promoting renewable energy sources in reducing environmental degradation, in Canada. When investigating the efficiency of energy use in some OECD countries (including Poland), Iram et al. (2020) showed that energy efficiency is crucial for environmental performance and carbon emission reduction. Moreover, as these authors pointed out, achieving an optimal level of energy efficiency should be prioritized over economic performance to improve environmental efficiency in these countries. In turn, Piwowar (2019) stated that, from the perspective of Polish agriculture, improving energy efficiency means, first of all, lowering fuel consumption. As we mentioned, production and use of fertilizers is strongly related to energy consumption. Therefore improving the efficiency of fertilizers (understood in our research as the amount and cost of fertilizers used) reduces energy use and promotes energy efficiency. According to Piwowar (2019), the use of fertilizers in Polish agriculture contributes to large emissions of nitrogen oxides. To increase fertilizer use efficiency, it is necessary to implement efficient, low-input agrotechnical practices. Hence, improvement of fertilizer efficiency use is an important task for achieving low-emission agriculture in Poland. Similar conclusions have been drawn in studies conducted in other countries. Xiong et al. (2021) proved that decreasing the total amount of fertilizers and increasing their efficiency are among the most important low-carbon technologies and management measures of high agricultural carbon productivity in Taihu Lake Basin, China. Koondhar et al. (2021b) proved that farmers in Pakistan should reduce chemical fertilizer application and increase the use of energy from renewable sources in order to increase grain food production, while reducing carbon emission. All these findings support the results obtained in our model.

In turn, the latent variable productivity is stimulated by the productivity of individual production factors (land, labor, and capital separately), as well as income and the share of agricultural income in the total income of a farmer’s household.

We should draw attention to the fact that labor productivity is determined by the value of land. Farmers who use agricultural land with higher market value may be more motivated to use labor more efficiently. This motivation can result from opportunity costs, which are very important at the microeconomic level, i.e., when a farmer is taking decisions about production processes. While we found that the amount of resources used in agricultural processes is not as crucial as we expected, we found that resources productivity plays a crucial role. The study shows that there is a strong association between our latent variables, low-carbon agriculture and resources productivity, and other variables, including income. Therefore, it is plausible to achieve both economic and environmental gains on a farm. Transforming production techniques as well as modernizing farming practices and introducing innovations are important in reducing the environmental footprint of agriculture. Such findings are supported by other researchers, who highlighted that promotion of best farming techniques, eco-innovation, and services that require capital are associated with improving environmental performance (Picazo-Tadeo et al. 2011; Van Grinsven et al. 2019).

There is a need to design and implement suitable institutional arrangements at the local, national, and EU levels. As Norse (2012) said, these are some key policy elements that should be considered for the global and domestic transition to low-carbon agriculture. They include (1) focusing on the largest sources of GHG with the lowest mitigation costs, (2) promoting multifunctional agriculture that achieves economic, environmental, and social objectives, and (3) prioritizing those measures that improve food security and are dedicated to poor groups, especially. Another important policy framework involves the substitution of conventional fossil energy with renewable energy, also in agriculture, including the European Union countries (Paramati et al. 2018). In turn, the great potential of renewable energy sources in reducing environmental pressure is described by Tillaguango et al. (2021) for Latin American countries and Oryani et al. (2021) in Iran. At the same time, the role of policy is also significant. Hamilton and Kelly (2017) opt for a multi-sector reform, which is important for the whole climate change policy, while Sharma et al. (2021b) suggested shaping a multipronged policy framework that allows to consider the need for complementarity between economic growth and environmental goals. Policymakers should promote cooperation with other countries and multinational corporations when introducing pro-environmental actions (Islam et al. 2021) and global economies are recommended to strive for integrating policies for emission reduction with the ones related to economic growth (Rehman et al. 2021). As summarized by Wiśniewski and Kistowski (2018), it is necessary to look at agriculture and rural areas more broadly when introducing low-carbon economy plans and strategies because agriculture (including forestry) has the potential to sequester carbon and reduce GHG emissions. According to Kampas et al. (2021), Poland may be seen as a country which is in line with the ecological modernization paradigm as Poland’s economic growth is not harmful to the environment or natural resources. In the context of patterns of CO_2_ emissions, Poland was compared to the Czech Republic, Germany, the Slovak Republic, and Sweden based on previous data and experiences (Narayan et al. 2016). Taking into account the abovementioned issues, low-carbon agriculture could definitely further contribute to this process.

## Managerial implications

Our findings imply that both income and resources productivity favor a low-carbon economy on a farm. This may be related to the fact that low-carbon farming requires capital expenditures: investing in thermal insulation of buildings, and buying more energy-efficient machinery, more precise fertilizers, and more efficient crop protection products. Based on research on GHG emissions in Polish agriculture, Kistowski and Wiśniewski (2020) stressed the need to design and use energy-efficient livestock buildings. In turn, Wan et al. (2013) addressed the issue of optimizing technology in order to move to low-carbon agriculture. However, this may be difficult for small farms with low incomes, because of the relatively higher costs and investments (Bonfiglio et al. 2017). As indicated by Ji et al. (2021) adopting the crop efficient production technique can yield the best ecological results. Policy incentives also are important. Gao (2019) demonstrated that households’ investment decisions are determined by their beliefs about gains. Therefore, we may conclude that financial incentives for farmers to implement low-carbon agriculture techniques could be considered.

Research findings show that there is an urgent need for education in the field of low-carbon economy. Access to up-to-date knowledge in this area and increased awareness should be considered as a kind of social investment. As Hudha et al. (2021) pointed out, it is all the more important as there are not enough issues related to low-carbon economy in primary-level textbooks. This problem should be prioritized both in school teaching and in advisory services for farmers. Hence, environmental issues are becoming increasingly important in farm management.

## Conclusion and policy implications

The idea of this article was to take an original approach in order to investigate low-carbon agriculture and contribute to existing research, as well as to identify the factors that promote the transition to low-carbon agriculture in the Wielkopolska region. The hypothesis that low-emission farming in the studies farms of Wielkopolska is determined by resource productivity was confirmed. The second hypothesis was proved as well. According to our research low-carbon agriculture is driven by growing energy efficiency and fertilizer efficiency. This indicates the importance of qualitative factors (i.e., production techniques and implementing innovations) in creating a low-carbon economy. Such new eco-friendly strategies involve investments in less energy-intensive machinery, thermal modernization of buildings, elimination of old types of furnaces, and the use of renewable energy sources (biogasification, photovoltaics). The latter are even more important as they are environmentally friendly and can be economically efficient.

Currently, the existing EU solutions (e.g., waste management, cross-compliance, greening, agri-environmental programs for environmental and animal welfare under the common agricultural policy, the 2007 Energy and Climate Package and the 2050 Roadmap) are, in our opinion, still insufficient to meet the global challenges of climate change and energy. Therefore, it is necessary to take complementary actions at the national and local levels to create incentives for developing a low-carbon economy. Experience in Poland with financial support for the installation of microphotovoltaic systems, thermal modernization of buildings, or replacement of central heating stoves is a good indication of this. It is also necessary to increase the awareness of farmers on these issues through education and training.

We believe that the issues of farm use of fertilizers and thermal insulation of buildings should be more broadly included in energy policy, both at the national and the European Union level. Some of these factors, however, are already present in the common agricultural policy for 2021–2027, namely, support for agricultural investments in environmental and climate protection (e.g., equipment and machinery for precise fertilizer application, investments in renewable energy sources in the production cycle, or improving energy efficiency of farm buildings used for agricultural production). In this way, the CAP is linked to energy policy.

Finally, it is also important to be aware of the limitations of this study as the research perspective is reduced. In particular, the availability of data that could be used to quantify the negative externalities associated with farm-level emissions is limited. One solution could be to gather more environmental indicators at the FADN level in the European Union countries. Further research on low-carbon economy, including low-carbon agriculture, should be even more integrated with climate change issues. In this context, such research should take into account the more detailed impact of individual countries and regions to incorporate this data into the assessment of emissions globally. It would be interesting to conduct similar research in regions with different levels of agricultural development. These studies should also include more qualitative variables in order to recognize the behavioral component of farmers’ decision-making in a low-carbon economy. In terms of research methods, applying structural equation modeling seems to be interesting and justified for such analysis.

## Data Availability

Data cannot be made public in the form of individual data because of obligations of secrecy. However, there is no restriction on their publication in aggregated and processed form.

## Footnotes

1 Economic size was defined as the standard value of agricultural output, known as the standard output (SO—the average monetary value of the agricultural output at the farm gate value of each agricultural product, crop, or livestock in a given region). It is expressed in thousands of EUR. The analysis used six classes of economic size: very small farms ES1 (EUR 2,000–8,000 SO), small farms ES2 (EUR 8,000–25,000 SO), medium ES3 (EUR 25,000–50,000 SO), medium–large ES4 (EUR 50,000–100,000 SO), large ES5 (EUR 100,000–500,000 SO), and very large ES6 (over EUR 500,000 SO).

2 The system distinguishes between eight types of production of agricultural holdings within the framework of the EU FADN agricultural accounting according to the predominant production type. These are as follows: field crops (TF1), horticulture (TF2), vineyards (TF3), other permanent crops (TF4), milk production (TF5), other grazing livestock (TF6), granivores (TF7), mixed (TF8).
